# Multiple Compact Camera Fluorescence Detector for Real-Time PCR Devices †

**DOI:** 10.3390/s21217013

**Published:** 2021-10-22

**Authors:** Seul-Bit-Na Koo, Hyeon-Gyu Chi, Jong-Dae Kim, Yu-Seop Kim, Ji-Sung Park, Chan-Young Park, Deuk-Ju Lee

**Affiliations:** 1 School of Software, Hallym University, Chuncheon-si 24252, Korea; rntmfqlcsk@gmail.com (S.-B.-N.K.); hyeongyuc96@gmail.com (H.-G.C.); kimjd@hallym.ac.kr (J.-D.K.); yskim@hallym.ac.kr (Y.-S.K.); 2 Bio-IT Research Center, Hallym University, Chuncheon-si 24252, Korea; 3 Biomedux, Suwon 16226, Korea; jspark@biomedux.com

**Keywords:** real-time PCR, fluorescence detection, open platform, image processing

## Abstract

The polymerase chain reaction is an important technique in biological research because it tests for diseases with a small amount of DNA. However, this process is time consuming and can lead to sample contamination. Recently, real-time PCR techniques have emerged which make it possible to monitor the amplification process for each cycle in real time. Existing camera-based systems that measure fluorescence after DNA amplification simultaneously process fluorescence excitation and emission for dozens of tubes. Therefore, there is a limit to the size, cost, and assembly of the optical element. In recent years, imaging devices for high-performance, open platforms have benefitted from significant innovations. In this paper, we propose a fluorescence detector for real-time PCR devices using an open platform camera. This system can reduce the cost, and can be miniaturized. To simplify the optical system, four low-cost, compact cameras were used. In addition, the field of view of the entire tube was minimized by dividing it into quadrants. An effective image processing method was used to compensate for the reduction in the signal-to-noise ratio. Using a reference fluorescence material, it was confirmed that the proposed system enables stable fluorescence detection according to the amount of DNA.

## 1. Introduction

The polymerase chain reaction (PCR) is an important technique in biological research because it can identify diseases with a small amount of DNA [1,2,3,4]. It is used in various fields such as infectious disease diagnosis, as well as for bacterial, viral, fungal and criminal investigations. In principle, PCR consists of a total of three steps, i.e., a denaturation step that separates the double-stranded DNA at high temperature, an annealing step that binds the primer to DNA, and an extension step that makes new DNA.

This three-step process is repeated to create new DNA. In general, PCR amplifies DNA and analyzes the results through several processes [5,6,7]. The DNA detection process comprises four steps: DNA extraction, DNA amplification, electrophoresis and gel image analysis. However, this process is time consuming and the analytical throughput is low. In addition, there is a high risk of false-positive results due to carryover contamination in the process of transferring amplification products for fluorescence detection and analysis.

To overcome these shortcomings, Real-Time PCR was developed. The amount of nucleic acid amplification products can be detected in real time, and the results can be analyzed in a shorter time than by conventional methods [8,9]. In addition, since there is no need to open the reaction tube, as was required in existing PCR experiments, the possibility of contamination is minimized.

There are two methods for detecting fluorescence in a general real-time PCR experiment: the first uses a photodiode and the second a camera [10,11]. To detect fluorescence, a dichroic mirror is usually used to form an optical unit for measurement. A dichroic mirror reflects light of one color and absorbs that of another. Compared to a general filter, there is an advantage in that the loss due to absorption is very small. However, it has a disadvantage in that it requires an optical distance to reflect and refract the light entering in a straight line. In addition, when designing a system, it takes a lot of time and money to purchase parts for each application, and to manufacture and use optical parts by hand. The other detection method, i.e., the camera-based method, is limited by its high cost and the large size of the detection device [12,13,14,15]. Camera-based devices for fluorescence detection mainly include large DSLR cameras; these devices are mostly used in hospitals or life science laboratories.

The open platform is evolving due to the development of smart communication technology and information technology [16,17]. Recently, many open platforms have been developed and used for image analysis after PCR detection. Images can also be easily analyzed using open software. High-performance smartphone cameras for open platforms are undergoing constant development. Accordingly, the field of imaging devices is also rapidly developing. With the development of CMOS sensor technology, manufacturing costs have been lowered, and miniature cameras with excellent performance which are suitable for portable devices are being developed.

LOC (Lab-on-a-Chip)-based technology is progressing due to developments in diagnostic devices [18,19]. Research in this field needs to be able to handle a small amount of sample, and it is important to reduce production costs. Therefore, the development of microfluidic channels has been ongoing. Channels that are easy to manufacture and which use flexible materials have been developed, and are available in various colors and thicknesses [20,21,22]. Although many research methods have been reported, most real-time PCR devices use a tube method that can detect many samples at once. Therefore, the use of real-time PCR equipment using tubes is common.

When measuring and analyzing fluorescence, the most important thing is to obtain accurate experimental results. To obtain accurate experimental results, images must be taken under the same conditions. Additionally, all tubes should be photographed and analyzed at the same time. To address this shortcoming, dozens of tubes are divided into several groups and photographed. Subsequently, several images are combined into a single image.

In this paper, we propose a low-cost and small-sized real-time PCR system including an open platform camera. The basic idea of the proposed system was presented at a conference, and this paper is an extended version of the presented content [23]. A case that blocks light was manufactured using parts that are widely available. A self-manufactured integrated board was used, and four low-cost, small cameras were attached for fixation. The whole tube was divided into quadrants and photographed with each camera. As a result, it was possible to minimize the field of view. An effective image processing method was used to compensate for the signal-to-noise ratio reduction of the superimposed image. To verify the proposed system, a comparative experiment was conducted using double distilled water and a reference fluorescence solution (FAM). The experimental results were verified by qualitative and quantitative analyses.

In our experiments, the system proposed in this paper was able to obtain similar results with a smaller size and lower cost than existing systems. Therefore, our system could be applied to various fluorescence measurement systems that require a wide field of view.

## 2. Materials and Methods

Table 1 shows the main components used in the experiment. In this paper, an ARDUINO Pro mini 328 5V and a SONY IMX 179 were used as the imaging systems. The ARDUINO Pro mini 328 has 14 digital input/output pins and 6 PWM pins. Its small size and low price make it useful for small product development and application. Table 2 shows an actual camera image and the specifications used in the experiments reported in this paper.

A filter based on green fluorescence emission was used for fluorescence measurements. The fluorescent reagent used was 6-FAM phosphoramidite (FAM). The excitation and emission filters were made of 5dia to fit the camera lens, making the optical system smaller and simpler.

**Table 2:** Camera image and specifications.

Classification	Detail
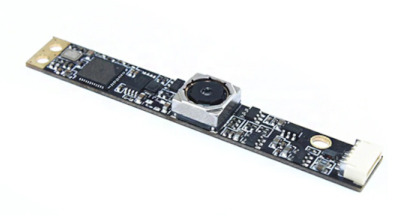	Optical Receiver: IMX 179 Focus: Auto Distance: 2 CM–100 M Viewing angle: Autofocus up to 75 degrees

**Figure 1:**
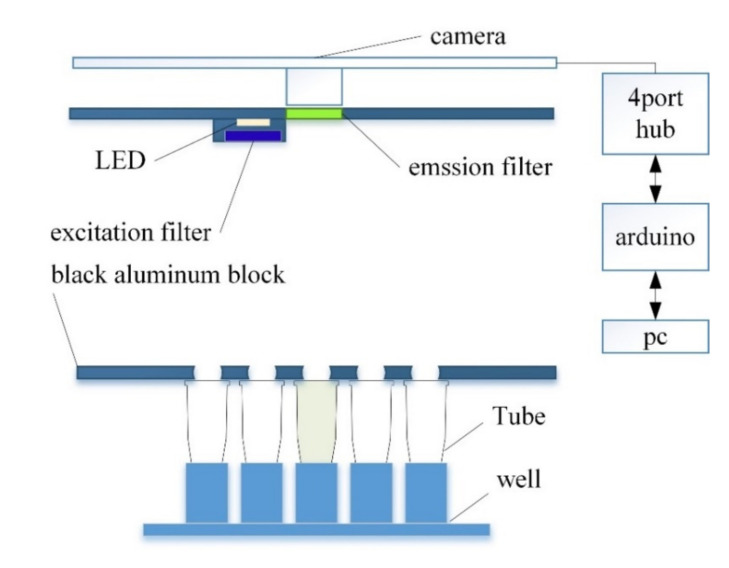
System structure diagram.

**Figure 2:**
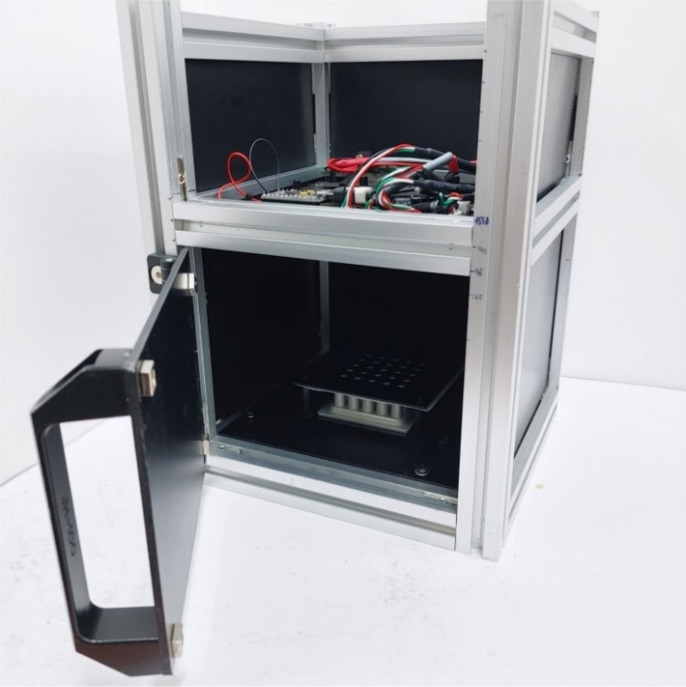
Proposed real-time PCR device.

Figure 1 shows the overall structure of the system proposed in this paper, and Figure 2 shows the actual real-time PCR device.

A dark room was constructed to prevent light blocking and reflections. The walls were made using matte acrylic, and the basic frame used an aluminum profile. To achieve a shooting distance of 100 mm, the height of the aluminum frame was 240 mm. At the bottom of the system is a block of matte black aluminum and 5 × 5 wells for holding 25 tubes.

An excitation filter was mounted on the front of the LED, and an emission filter was mounted on the front of the camera to measure the fluorescence reflected by the tube. The LED and camera are connected to the Arduino through a hub so that they are turned on only during shooting to prevent the LED light from changing. The minimum focal length can be fine-tuned by adjusting the height between the tube and the camera, such that the same fluorescence detection was consistently possible.

**Figure 3:**
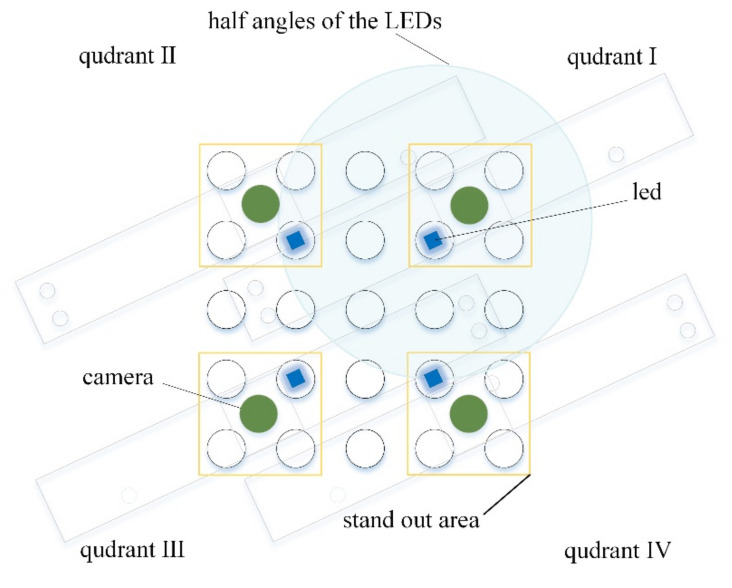
Quadrant of all 5 × 5 wells: camera and LED location.

Figure 3 shows the position of the LED and camera required to image the entire tube under the same conditions.

**Figure 4:**
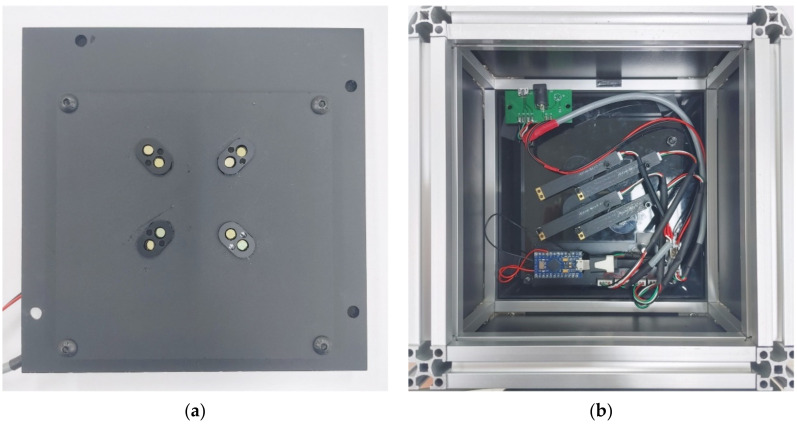
(**a**) Front of the imaging system; (**b**) Back of the imaging system.

Figure 3 also shows the range of quadrant that each camera can capture. The green circles represent the four camera positions and the blue squares the four LED positions. In order to minimize the optically complex environment, the camera and LED were arranged so that the 5 × 5 well could be divided into 3 × 3 quadrants. Yellow boxes show the stand out areas in each quadrant when shot with each camera. Due to this structure and arrangement, the entire tube can be viewed uniformly from all angles, and an image with low distortion can be obtained. Figure 4 shows the front and back of the imaging system board.

**Figure 5:**
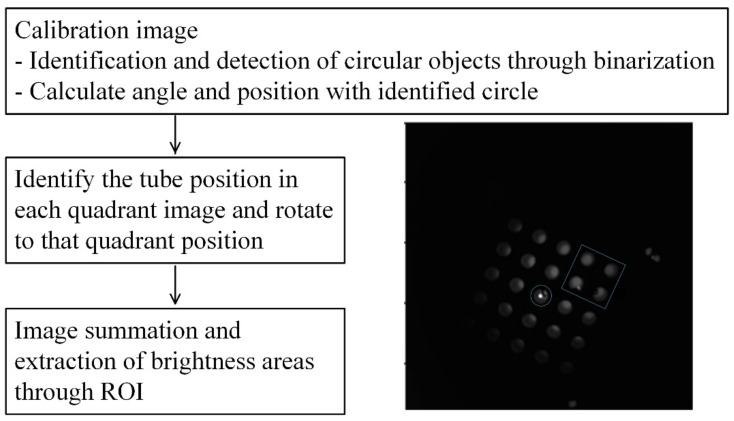
Imaging process diagram.

Figure 5 shows an image processing diagram and the center of gravity and bright area in the first quadrant. In order to find the most stable position of the tube photographed in each quadrant, a fluorescent mark sheet for easy image processing was made and attached to the bottom of the aluminum well. For calibration, each quadrant was photographed to identify 25 circles, after which the positions of the circles and rotation angles in each quadrant were extracted. The center and angle of image rotation were calculated at the 5 × 5 tube center and the position of the tube with the largest *X*-axis value.

For PCR emulation, 0.14 µmol/μL of FAM solution was used. The solution had the same fluorescence brightness as that measured when DNA has been fully amplified. FAM solutions were added to 25 tubes of 36 μL each, and inserted into 5 × 5 wells placed in order to obtain four quadrant images. DDW also acquired four quadrant images using 25 tubes containing 36 μL. 

**Figure 6:**
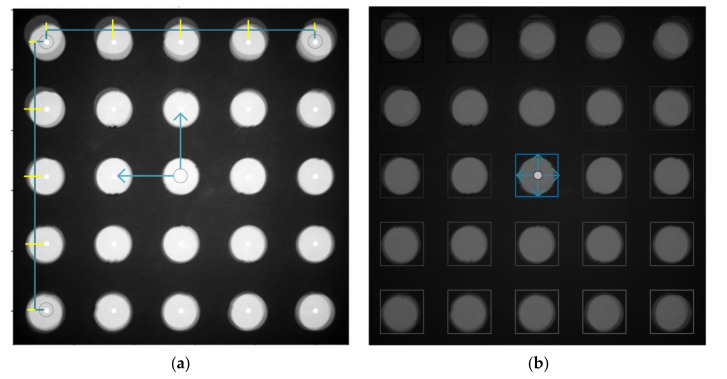
Fluorescence brightness extraction process of the merged image: (**a**) Extraction of 25 tube center positions by bilinear interpolation. (**b**) ROI of each tube.

The four images obtained for each solution were combined into a single image based on information obtained during the calibration of each quadrant. The quadrant image identifies the tube in its unique circle position in each quadrant and rotates the image through the angle of rotation. After rotation, images are cut to the same size, and positioned in the center of the tube in quadrant to fit one image. Figure 6 shows the process of extracting brightness after image matching. Figure 6a shows the center position of each tube in a 5 × 5 array marked with a white dot. By using the bilinear interpolation method through the centers of the four corner tubes, the positions of the centers of the remaining tubes can be found. Since the image distortion of the proposed system is very small, it can be assumed that the interval obtained by dividing the width and height from the centers of the corner tube into quarters is the same as the tube spacing. The blue rectangles in Figure 6b show the ROI (region of interest) of each tube. Since the tube, ROI and center may become misaligned for each experiment, a brightness average was obtained in a square area that was significantly smaller than the tube diameter, such that the area in which to obtain the average brightness was always located in the tube. In this study, the size of the entire ROI area was 161 × 161, and the square area to obtain the average brightness was set to 80 pixels per side.

## 3. Results

**Figure 7:**
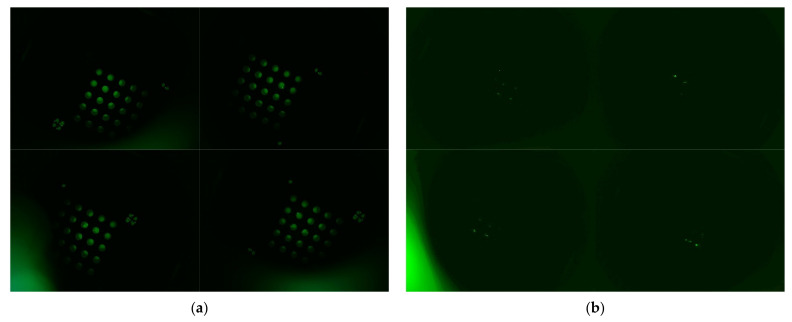
Four images divided into quadrants: (**a**) FAM; (**b**) DDW (Increased by a factor of six).

Figure 7 shows each image taken by dividing it into quadrants. The image on the left was taken from 25 tubes containing 0.14 μmol/μL of FAM solution. A solution of 0.14 μmol/μL of FAM matches the fluorescence brightness of a solution in which the DNA has been completely amplified. The right side of Figure 7 shows images taken from 25 tubes containing DDW. The DDW image was amplified six times to improve visibility.

Since the cameras in the quadrant are fixed, the position of the imaged tube corresponding to each quadrant will always be the same. The positions of the tubes in the four images are different. To synthesize one image, the position and rotation angle of the tube must be calculated.

**Figure 8:**
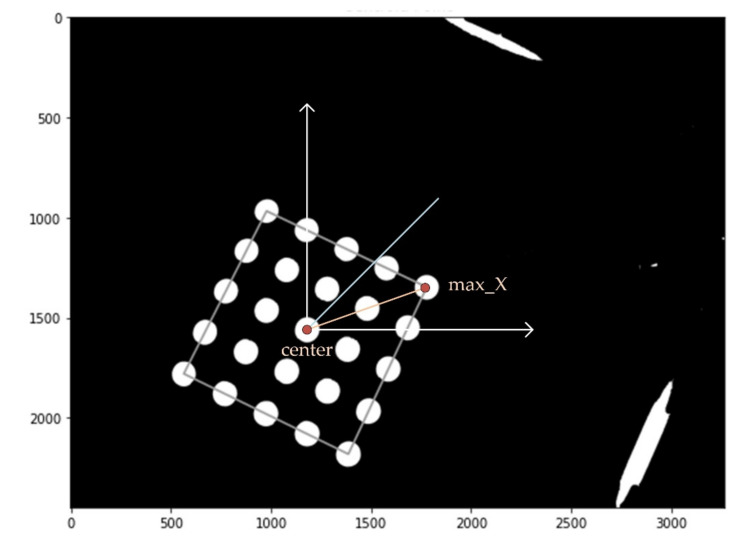
Method of determining the center and rotation angle of each quadrant image.

Figure 8 shows the direction in which the center of the tube array and the tubes in row 1 and column 5 are connected in the image of the first quadrant (yellow vectors). If the image is rotated counterclockwise so that the yellow and blue vectors are aligned, the rows and columns of the tube array are also aligned with their coordinate axes (white vector). If the four quadrant images are rotated in this way, each tube is located in the same place. The images in the first and second quadrants are rotated counterclockwise by about 25°, and those in the third and fourth by about 135°.

**Figure 9:**
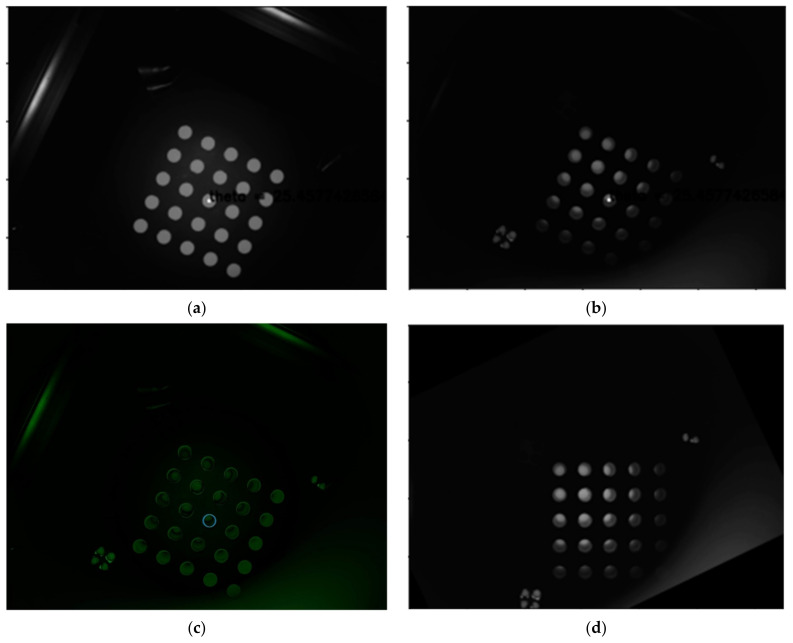
(**a**) Mask sheet for calibration; (**b**) FAM reagent tubes; (**c**) Image of the difference; (**d**) Rotated image.

Figure 9a is a second quadrant image taken using a mask sheet, and Figure 9b is a second quadrant image taken of a tube containing a FAM reagent. Figure 9c illustrates the difference between the mask sheet and the fam solution by combining them. When the position of the diameter of the circle at the center of the 5 × 5 tube was marked, the distances between the tubes comprised fewer than 1.5 pixels. Therefore, it was found that there was no problem in using the image rotation center and rotation angle calculated by the mask sheet. Figure 9d shows the image rotated by the rotation center and rotation angle obtained with the mask sheet in the FAM image of (b). This image also showed that the calibration was stable.

**Figure 10:**
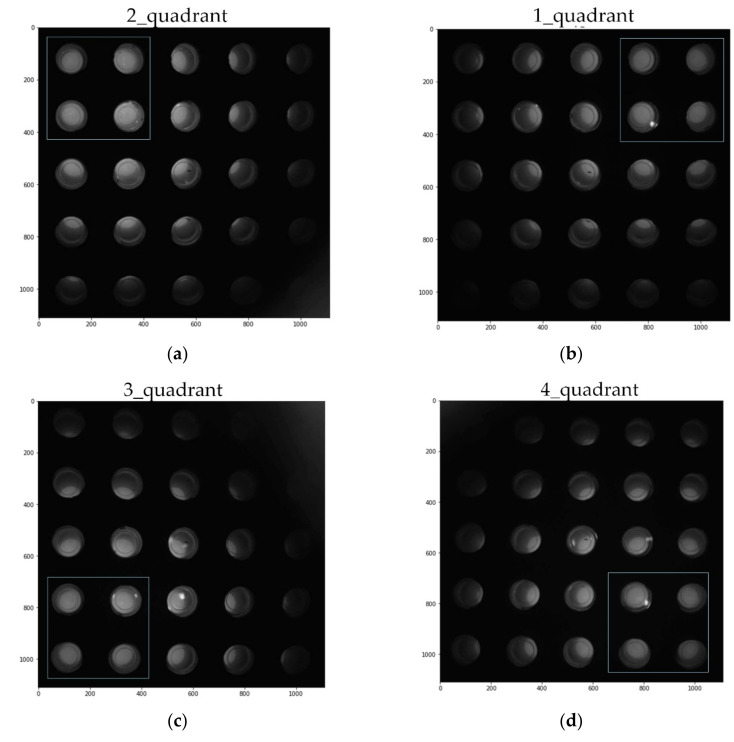
Image cropped to the same size in the center (FAM): (**a**) 2 quadrant; (**b**) 1 quadrant; (**c**) 3 quadrant; (**d**) 4 quadrant.

Figure 10 is an image cut to the same size after adding fam reagent and rotating the images taken for each quadrant counterclockwise. When it is rotated, it looks like Figure 9d, but since it has information about the center of gravity of each quadrant, it is possible to obtain a 5 × 5 well image of a constant array by cutting it to the same size. A relatively bright 2 × 2 tube is shown positioned on the bright side of the corresponding quadrant.

**Figure 11:**
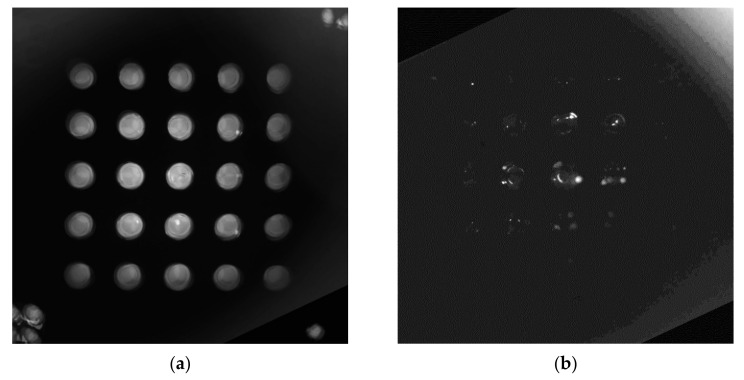
Image of combined from each quadrant into one: (**a**) FAM; (**b**) DDW (Increase by a factor of six).

Figure 11 shows an image obtained by synthesizing the images of each quadrant into a single image. Figure 11a is a fam image, and Figure 11b shows a DDW image. As mentioned earlier, we increased the image brightness of the DDW by a factor of six. The two images show an ideal match.

**Figure 12:**
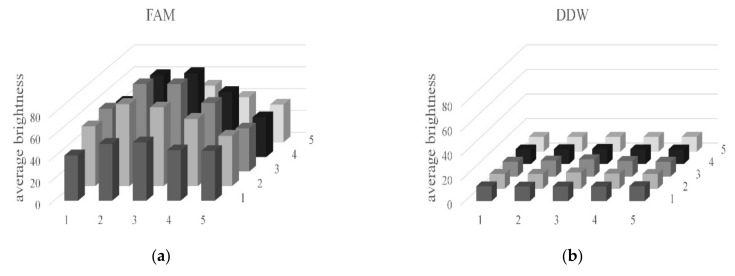
Average brightness of the tubes with: (**a**) FAM; (**b**) DDW.

Figure 12 shows the average brightness of each of the 25 tubes. The graph on the left is an image of a tube containing FAM, and that on the right a tube containing DDW.

**Figure 13:**
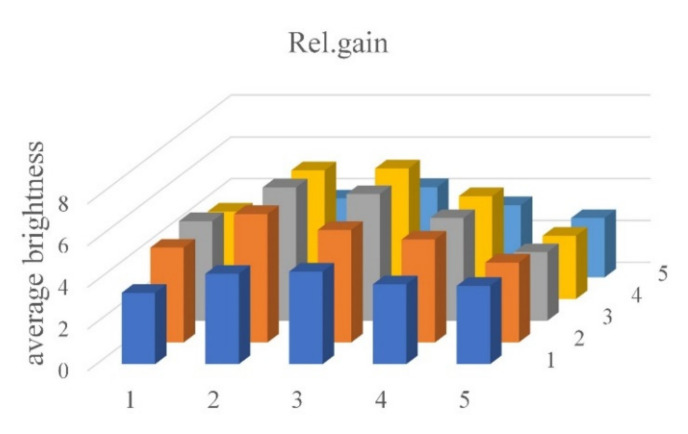
Relative gains.

The average brightness of the tube image containing DDW was about 12. The average brightness of the tube image containing FAM was generally 55 or higher. Figure 13 shows the relative gain difference between the FAM and the DDW images. In fluorescence detection, brightness through relative gain is based on tubes in the same location in each well, i.e., it is the value obtained by dividing the brightness of the DDW image by the difference between the brightness of FAM and DDW images. It is generally more than 2.84, as shown in Table 3. Table 3 shows the average brightness and relative gain of tubes containing FAM and DDW.

## 4. Conclusions and Discussion

In this paper, we propose a fluorescence measurement device for low-cost, compact real-time PCR using an open platform camera. In order to obtain a wide field of view, four low-cost cameras with a small field of view were used. We propose a method to capture all of each of the wells using four cameras. Areas with weak fluorescence were able to obtain sufficient brightness by superimposing quadrant images, and were corrected through image processing. Experimental results were obtained using DDW and FAM with fully amplified DNA brightness. As a result, the relative gain was more than 2.84 in all wells. 

Therefore, it was concluded that the fluorescence detector for real-time PCR devices proposed in this paper is sufficient for fluorescence detection. The method proposed in this paper is expected to reduce the cost and size of such optical systems.

This paper described an experiment with a novel fluorescence detector system that could be applied to real-time PCR. In future research, based on the results presented in the present study, a prototype including heating and cooling elements will be completed. We also plan to make several prototypes and conduct performance evaluations. Important experimental elements for performance evaluation are standard DNA tests and real sample tests. A performance evaluation comparing a commercial system and the completed prototype system should also be performed. It is expected that good results will be obtained through these comparative experiments.

## Figures and Tables

**Table 1:** System components.

Classification	Detail	
Imaging System	IMX 179 Sony sensor 77.2° view-angle	
Fluorescence reagent	Probe type FAM (0.14 μmol/μL)	
Control reagent	DDW (Double Distilled Water)	
Filter	Excitation	CW: 470 nm, BW: 40 nm
	Emission	CW: 535 nm, BW: 50 nm
Light	White Led (240 mA, 120° view-angle)	

**Table 3:** Brightness and relative gain statistics of each tube.

	FAM	DDW	Rel. Gain
mean	55.23	12.27	4.48
min	34.48	12.00	2.84
max	85.11	14.03	6.37

## Data Availability

Data sharing is not applicable to this article.

## References

[1] Koo C., Malapi-Wight M., Kim H.S., Cifci O.S., Vaughn-Diaz V.L., Ma B., Kim S., Abdel-Raziq H., Ong K., Jo Y.-K. (2013). Development of a real-time microchip PCR system for portable plant disease diagnosis. PLoS ONE.

[2] Zhang C., Xing D. (2007). Miniaturized PCR chips for nucleic acid amplification and analysis: Latest advances and future trends. Nucleic Acids Res..

[3] Zhang C., Xing D., Li Y. (2007). Micropumps, microvalves, and micromixers within PCR microfluidic chips: Advances and trends. Biotechnol. Adv..

[4] Chan-Young P., Mi-So L., Yu-Seop K., Song H.-J., Jong-Dae K. (2018). Sensor data abstraction for failure prediction of polymerase chain reaction thermal cyclers. Int. J. Eng. Technol. Innov..

[5] Salm E., Liu Y.-S., Marchwiany D., Morisette D., He Y., Bhunia A.K., Bashir R. (2011). Electrical detection of dsDNA and polymerase chain reaction amplification. Biomed. Microdevices.

[6] Wu J., Kodzius R., Xiao K., Qin J., Wen W. (2012). Fast detection of genetic information by an optimized PCR in an interchangeable chip. Biomed. Microdevices.

[7] Kodzius R., Xiao K., Wu J., Yi X., Gong X., Foulds I.G., Wen W. (2012). Inhibitory effect of common microfluidic materials on PCR outcome. Sens. Actuators B Chem..

[8] Hwang J.-S., Kim Y.-S., Song H.-J., Kim J.-D., Park C.-Y. (2016). Fluorescence detection test by black printed circuit board based microfluidic channel for polymerase chain reaction. Technol. Health Care.

[9] Cikos S., Koppel J. (2008). Transformation of real-time PCR fluorescence data to target gene quantity. Anal. Biochem..

[10] Xiang Q., Xu B., Li D. (2007). Miniature real time PCR on chip with multi-channel fiber optical fluorescence detection module. Biomed. Microdevices.

[11] Sun K., Yamaguchi A., Ishida Y., Matsuo S., Misawa H. (2002). A heater-integrated transparent microchannel chip for continuous-flow PCR. Sens. Actuators B Chem..

[12] Lee D.-J., Kim S.-Y., Kim J.-D., Kim Y.-S., Song H.-J., Park C.-Y. (2013). Low-cost gel imaging system implementation in reduced size. Int. J. Bio-Sci. Bio-Technol..

[13] Goldmann T., Zyzik A., Loeschke S., Lindsay W., Vollmer E. (2001). Cost-effective gel documentation using a web-cam. J. Biochem. Biophys. Methods.

[14] Porch T.G., Erpelding J.E. (2006). Low-cost conversion of the Polaroid MD-4 land camera to a digital gel documentation system. J. Biochem. Biophys. Methods.

[15] Mendoza-Gallegos R.A., Rios A., Garcia-Cordero J.L. (2018). An affordable and portable thermocycler for real-time PCR made of 3D-printed parts and off-the-shelf electronics. Anal. Chem..

[16] Vujovic V., Maksimović M., Vujović V., Davidović N., Milošević V., Perišić B. (2014). Raspberry Pi as Internet of Things hardware: Performances and Constraints Raspberry Pi as Internet of Things hardware: Performances and Constraints. Des. Issues.

[17] Wilkes T.C., McGonigle A.J., Pering T.D., Taggart A.J., White B.S., Bryant R.G., Willmott J.R. (2016). Ultraviolet imaging with low cost smartphone sensors: Development and application of a raspberry Pi-based UV camera. Sensors.

[18] Neuzil P., Pipper J., Hsieh T.M. (2006). Disposable real-time microPCR device: Lab-on-a-chip at a low cost. Mol. Biosyst..

[19] Ahrberg C.D., Ilic B.R., Manz A., Neužil P. (2016). Handheld real-time PCR device. Lab A Chip.

[20] Kim J., Byun D., Mauk M.G., Bau H.H. (2009). A disposable, self-contained PCR chip. Lab A Chip.

[21] Shen K., Chen X., Guo M., Cheng J. (2005). A microchip-based PCR device using flexible printed circuit technology. Sens. Actuators B Chem..

[22] Moschou D., Vourdas N., Kokkoris G., Papadakis G., Parthenios J., Chatzandroulis S., Tserepi A. (2014). All-plastic, low-power, disposable, continuous-flow PCR chip with integrated microheaters for rapid DNA amplification. Sens. Actuators B Chem..

[23] Koo S.-B.-N., Chi H.-G., Park J.-S., Kim J.-D., Park C.-Y., Kim Y.-S., Lee D.-J. (2021). Multiple Camera Fluorescence Detection for Real-Time PCR. Eng. Proc..

